# The dynamic landscape of T helper cell plasticity and Th17–Treg instability in periodontitis

**DOI:** 10.1038/s12276-026-01847-4

**Published:** 2026-10-01

**Authors:** Yun-Ji Lim, Tae Sung Kim

**Affiliations:** 1 Department of Biochemistry, School of Medicine Pusan National University, Yangsan, Republic of Korea; 2 Research Institute for Convergence of Biomedical Science and Technology, Pusan National University Yangsan Hospital, Yangsan, Republic of Korea; 3 Department of Oral Microbiology, School of Dentistry, Dental and Life Science Institute, Pusan National University, Yangsan, Republic of Korea; 4 Education and Research Team for Life Science on Dentistry, Pusan National University, Yangsan, Republic of Korea

**Keywords:** Mucosal immunology, Chronic inflammation

## Abstract

Periodontitis is a chronic inflammatory disease initiated by a dysbiotic microbiome, yet tissue destruction is primarily driven by an exaggerated host immune response. CD4⁺ T helper (Th) cell subsets play important roles in regulating periodontal inflammation and osteoclast-mediated bone resorption through distinct cytokine networks. Th1 and Th17 responses are closely associated with pro-inflammatory signaling and alveolar bone loss, whereas Th2 and regulatory T (Treg) cells exert context-dependent immunomodulatory functions. Beyond these subsets, Th9 and Th22 cells further expand the complexity of T cell-mediated regulation in periodontal lesions. Importantly, accumulating evidence supports T helper cell plasticity, exemplified by the emergence of IL-17⁺ Treg, suggesting that periodontal disease reflects immune imbalance and functional instability rather than the dominance of a single lineage. Clarifying these mechanisms may support the development of targeted immunomodulatory strategies aimed at restoring oral mucosal immune homeostasis. This review shifts the focus from static T cell subsets to their dynamic phenotypic flexibility, highlighting how maladaptive transitions drive chronic tissue destruction.

## T cell plasticity drives periodontitis immune imbalance

Periodontitis, a chronic inflammatory disease, involves the progressive destruction of tooth-supporting structures due to dysregulated immune responses rather than direct bacterial toxicity. This review highlights the complex interplay of CD4⁺ T cell subsets, such as T helper 1 (Th1), Th17, and regulatory T (Treg) cells, in periodontal inflammation. The authors emphasize T cell plasticity, the Th17–Treg imbalance, and the functional instability of Treg as key mechanisms driving tissue destruction. Emerging hybrid T cell populations further illustrate how phenotypic instability contributes to chronic inflammation and bone loss. Understanding dynamic T cell interactions within the periodontal microenvironment may guide targeted therapies. Future research should focus on reinforcing regulatory stability and restoring immune homeostasis to prevent bone loss in periodontitis.

This summary was initially drafted using artificial intelligence, then revised and fact-checked by the author.

## Introduction

Periodontitis is a prevalent chronic inflammatory disease characterized by progressive destruction of the tooth-supporting structures, including the gingiva, periodontal ligament, and alveolar bone^1,2^. It is increasingly recognized as a multifactorial disorder driven by complex and interrelated pathophysiological processes^3^. Disease initiation and progression arise from dynamic interactions among environmental factors (e.g., smoking and stress), dysbiotic microbial communities (e.g., *Porphyromonas gingivalis*, *Treponema denticola*, *Tannerella forsythia*, and *Aggregatibacter actinomycetemcomitans*), and dysregulated host inflammatory and immune responses^4–8^. Although periodontitis is triggered by a dysbiotic microbiome, accumulating evidence indicates that disease progression and tissue destruction are governed primarily by dysregulated host immune responses rather than by direct bacterial toxicity^6,9^. Connective tissue breakdown and inflammatory bone resorption stem from the sustained activation of host inflammatory and osteoclastogenic networks, highlighting that periodontitis is best conceptualized as a chronic immunopathological condition rooted in impaired immune homeostasis^10,11^. The transition from microbial colonization to destructive oral mucosal inflammation involves a complex interplay between innate immune activation and adaptive immune regulation within the periodontal microenvironment^12,13^.

Among adaptive immune components, CD4⁺ T cells have emerged as the pivotal regulators of oral mucosal immunopathology^13^. Upon activation by antigen-presenting cells and cytokine cues, naïve CD4⁺ T cells differentiate into functionally specialized subsets, including T helper 1 (Th1), Th2, Th9, Th17, Th22, and regulatory T (Treg) cells^14,15^. Each subset is characterized by distinct transcription factors and cytokine profiles that collectively shape local immune responses. Historically, periodontal disease was interpreted through a simplified Th1–Th2 paradigm, in which Th1 responses were associated with pro-inflammatory tissue destruction and Th2 responses were consisdered anti-inflammatory or humoral immune functions^16,17^. However, advances in immunology have revealed a far more intricate immune landscape involving additional T helper subsets and dynamic cross-regulatory mechanisms^13,18,19^.

In particular, Th17 cells have been identified as potent drivers of osteoclastogenesis and inflammatory bone loss^20^, whereas Treg cells play critical roles in limiting excessive inflammation and maintaining immune homeostasis^21^. Emerging subsets, such as Th9, Th22, and T follicular helper (Tfh) cells, further expand the complexity of T cell-mediated immune regulation in chronic inflammatory diseases^13,22–24^; however, their precise mechanistic contributions to periodontal pathology remain incompletely defined. Importantly, increasing evidence supports the concept of T helper cell plasticity, whereby CD4⁺ T cells exhibit functional flexibility and may adopt hybrid phenotypes under inflammatory conditions. This plasticity challenges the notion of rigid lineage commitment and underscores the dynamic nature of immune regulation in chronic periodontal inflammation.

T cell plasticity refers to the capacity of differentiated CD4⁺ T cells to acquire alternative functional or phenotypic characteristics in response to environmental cues^25^. Phenotypic instability refers to the loss or alteration of lineage-defining features, whereas functional flexibility refers to context-dependent changes in effector functions without complete lineage conversion^26^. Together, these processes reflect immune adaptation, whereby T cell responses are dynamically recalibrated according to the local inflammatory microenvironment.

Given these evolving insights, a comprehensive understanding of CD4⁺ T cell subset interactions, imbalance, and phenotypic instability is essential for redefining periodontal disease not merely as a bacterial infection, but as an immune-mediated inflammatory disorder. Although T cell subsets have been broadly identified, how their phenotypic flexibility and spatial organization within gingival tissues drive chronic destruction remains a critical knowledge gap. Unlike previous descriptive reviews focused primarily on static T helper cell classifications, this review emphasizes T cell plasticity, functional instability, and spatial immune organization as central mechanisms underlying chronic periodontal inflammation and tissue destruction. We aim to examine how T cell plasticity, functional instability, and spatial immune organization collectively govern the transition from protective to destructive immune responses in periodontitis. The functional characteristics, inducing signals, and the strength of mechanistic evidence for each T helper cell subset are systematically summarized in Table 1.

**Table 1:** Systematic comparison of T helper cell subsets in periodontitis.

Subset	Inducing signals and cellular sources	Transcription factors and markers	Signature cytokines	Effects on immune and non-immune cells	Strength of mechanistic evidence
**Th1**	IL-12 and IL-18 provided by APCs^41^; pathogen-derived lipopolysaccharide ^27,28^	T-bet^27,28^	IFNγ^27,28^	Modulates macrophage activation and pro-inflammatory signaling^28^; stimulates MMP secretion^38,42^; exhibits complex effects on osteoclastogenesis, ranging from in vitro inhibition to in vivo correlation with bone resorption^30,31^	Mechanistic and causal evidence (experimental models)^30,31^
**Th2**	IL-33 from gingival epithelial cells, fibroblasts, and endothelial cells	GATA3, ST2^45,53,54^	IL-4, IL-13^34,43,44^	Inhibits pro-inflammatory drivers (IL-1, TNFα, IL-6)^46^; upregulates TGFβ; IL-13 specifically downregulates MMP1 in gingival fibroblasts^49^; supports humoral immunity^34^	Primarily correlative evidence (clinical studies)^46,47^
**Th9**	Combined signaling of IL-4 and TGFβ^15^	PU.1, IRF4 (inferred from general immune context)^22^	IL-9, IL-10, IL-21^15,22^	Modulates mast cell degranulation and neutrophil chemotaxis^58^; associated with inflammatory activity and tissue remodeling in periodontal lesions^59,60^	Primarily correlative evidence (clinical studies)^59,60^
**Th17**	Microbiome-driven IL-1β, IL-6, and IL-23 from APCs, with epithelial IL-23 signaling^61,62,70–72^	RORγt^15,61,63^	IL-17A, IL-17F, IL-21, IL-22^15,61^	Increases RANKL/OPG ratio in osteoblasts and PDL cells^32,62,65^; recruits neutrophils via CXCL1, CXCL2, and G-CSF^19^; promotes MMP, ROS, and NET production^66–68,71^	Strong mechanistic and causal evidence (experimental expansion–depletion models)^32,64^
**Th22**	Overlapping inflammatory milieu with Th17 (IL-6, TNFα, IL-23)^86,87^	Spi-B, AhR^86,87^	IL-22^81,82^	Correlates with elevated RANKL and alveolar bone loss^86,87^; contributes to mineralized matrix-forming repair activities in periapical sites^83,85^	Partial mechanistic evidence (animal models)^87^
**Tfh**	IL-6 and IL-21 derived from APCs and activated T cells^15,89^	Bcl6, CXCR5, ICOS, PD-1^83,88,89^	IL-21^64,90,91^	Promotes B cell activation, germinal center formation, and antibody production^90–92^; possible Th17–Tfh interplay^93^	Primarily correlative evidence (clinical studies)^83,92,93^
**Treg**	TGFβ^15,96,97^	Foxp3, CD25, CD127^low^^15,94,95,101^	TGFβ, IL-10, IL-35^15,96,97^	Restrains Th1-, Th17-, and B cell-mediated inflammation^88,96,98^; indirectly attenuates RANKL expression and suppresses osteoclastogenesis^89,101,102^	Mechanistic and causal evidence (experimental expansion–depletion models)^104,105^

Table 1 synthesizes the functional diversity of CD4⁺ T helper cell lineages within the periodontal microenvironment, emphasizing the interplay between specific inducing signals, transcriptional regulation, and their subsequent impact on tissue homeostasis and alveolar bone stability. *AhR* aryl hydrocarbon receptor, *APCs* antigen-presenting cells, *Bcl6* B cell lymphoma 6, *Foxp3* forkhead box P3, *GATA3* GATA-binding protein 3, *G-CSF* granulocyte colony-stimulating factor, *IFNγ* interferon-γ, *IL* interleukin, *ILC3* group 3 innate lymphoid cell, *IRF4* interferon regulatory factor 4, *MMP* matrix metalloproteinase, *OPG* osteoprotegerin, *PU.1* Spi-1 proto-oncogene, *RANKL* receptor activator of nuclear factor-κB ligand, *RORγt* retinoic acid receptor-related orphan receptor gamma t, *STAT3* signal transducer and activator of transcription 3, *Tfh* T follicular helper cells, *TGFβ* transforming growth factor-β, *TNFα* tumor necrosis factor-α, *Treg* regulatory T cells.

## Th1 cells

In periodontal disease, Th1 cells are a subset of CD4⁺ T lymphocytes that contribute to the adaptive immune response against periodontal pathogens within gingival tissues. In periodontal lesions, innate immune signals from pathogen-associated molecular patterns such as bacterial lipopolysaccharide and cytokines (e.g., IL-12) promote Th1 differentiation, leading to increased production of interferon-γ (IFNγ) and other pro-inflammatory mediators^27,28^.

Th1 cells produce IFNγ, which plays an important role in periodontal tissues. First, IFNγ participates in macrophage activation and pro-inflammatory signaling. Activated macrophages produce additional pro-inflammatory cytokines and enzymes to control microbial challenge. However, excessive or chronic activation can also contribute to tissue inflammation and matrix breakdown in periodontitis^28^. Several clinical and experimental studies have reported elevated levels of Th1 cells and IFNγ in patients with periodontitis compared with healthy controls. For example, patients with periodontitis exhibit higher proportions of circulating and local Th1 cells and increased IFNγ expression compared with controls^29^, suggesting that Th1-mediated immune responses are associated with disease presence and severity.

However, the role of Th1-driven IFNγ in periodontal pathology is complex and remains debated. Some evidence suggests that IFNγ suppresses osteoclast differentiation in vitro, thereby protecting against bone loss, whereas other studies indicate that Th1-biased responses and IFNγ production correlate with increased inflammatory bone resorption in periodontal lesions^30,31^. This complexity likely reflects the balance between protective antibacterial responses and host-mediated tissue damage during chronic inflammation. Importantly, IFNγ plays a context-dependent role in periodontitis^32^. Although IFNγ promotes inflammatory activation of macrophages and enhances local cytokine production^33,34^, it can also directly inhibit osteoclast differentiation under certain conditions^35^. Thus, the net effect of Th1 responses likely depends on the balance between antimicrobial defense, inflammatory amplification, and regulation of bone remodeling.

IL-18, a pro-inflammatory cytokine originally identified as an IFNγ-inducing factor, is increasingly recognized as an important mediator in periodontal pathogenesis^36^. Clinical studies consistently report that IL-18 levels are notably elevated in the serum, saliva, and gingival crevicular fluid (GCF) of patients with periodontitis^37–40^. Importantly, IL-18 acts synergistically with IL-12 to promote IFNγ production in the Th1 response^41^, sustaining Th1-associated immune activity within periodontal lesions. Therefore, the IL-18–Th1 axis may represent an important immunological link in understanding the pathophysiology of periodontal disease. Beyond amplifying Th1-associated immune responses, IL-18 may also directly participate in periodontal tissue destruction. IL-18 reportedly stimulates the activation of matrix metalloproteinase (MMP), leading to inflammatory bone loss after oral bacterial infection (*P. gingivalis*)^38,42^.

Thus, in periodontal disease, Th1 cells and their signature cytokine IFNγ play an important role in shaping the local immune environment by activating macrophages, amplifying pro-inflammatory responses, and participating in mechanisms that link adaptive immunity to periodontal tissue destruction and alveolar bone loss. Recent studies further suggest that Th1 responses in periodontitis are integrated within broader inflammatory networks involving Th17-associated cytokines and signaling pathways^18,32^. Although direct evidence for Th1–Th17 lineage interconversion in periodontitis remains limited, the concurrent presence of Th1- and Th17-associated responses highlights the dynamic and interconnected nature of T helper cell regulation during chronic periodontal inflammation^29^.

## Th2 cells

Th2 cells, characterized by the master transcription factor GATA3 and secretion of IL-4 and IL-13, have been identified in periodontal tissues^34,43–45^. These cytokines are associated with humoral immunity and B cell activation, and are part of the broader T helper cell response detected in periodontitis^34^. Notably, Th2 cells possess immunomodulatory functions; for instance, IL-4 can inhibit the expression of key pro-inflammatory cytokines, including IL-1, tumor necrosis factor-α (TNFα), and IL-6, suggesting a role in the regulation of periodontal inflammation^46^. Clinical studies have demonstrated that GCF IL-4 concentrations are notably lower in periodontitis sites than in healthy gingiva^46,47^, indicating that IL-4 may be associated with the resolution or remission of inflammation.

IL-13 shares many functions with IL-4, including inhibition of macrophage activation and support for anti-inflammatory states, which could limit bone resorption under certain conditions^48^. IL-13 can upregulate transforming growth factor-β (TGFβ) and downregulate collagen‑degrading MMP1 in gingival fibroblasts^49^, suggesting a protective role of Th2 cytokines by suppressing pro-inflammatory cytokine production against excessive tissue destruction in periodontitis^27^.

In addition, IL-33 expression has been detected in gingival epithelial cells, fibroblasts, and endothelial cells, and is notably elevated in inflamed gingival tissues and GCF in periodontal disease^50–52^. IL-33 binds to expressed ST2 (IL-33 receptor) on Th2 lymphocytes and acts as a chemoattractant for these cells^53^. Notably, IL-33 shapes a Th2-skewed immune microenvironment within periodontal lesions, and acts as a potent inducer of Th2-associated cytokines^27,53,54^. Although IL-33-driven Th2 cytokines, particularly IL-4 and IL-13, exert immunomodulatory effects that limit tissue destruction^52^, some studies suggest that IL-33 also amplifies local inflammation by enhancing early inflammatory responses and exacerbating bone loss^51,55^. IL‑33 is typically an epithelial‑derived alarmin; depending on whether it is released in controlled or necrotic epithelial contexts, its downstream Th2 effects differ. The Th2–IL-33 axis likely plays a dual and context-dependent role in periodontal disease; however, this axis may therefore represent an important immunological mechanism involved in the modulation of periodontal inflammation and disease progression, and could serve as a potential target for host modulation strategies in periodontal therapy.

Overall, Th2-associated cytokines, including IL-4, IL-13, and IL-33, are increasingly recognized as important modulators of periodontal immune responses. Rather than serving as primary drivers of tissue destruction, Th2 responses function as potential immunoregulatory pathways that influence periodontal disease progression by balancing inflammation and tissue protection in a disease stage- and immune context-dependent manner^16^. Importantly, the relative contribution of Th2-associated responses may vary depending on the inflammatory stage and local cytokine milieu, highlighting the context-dependent nature of T helper cell regulation within periodontal lesions^44,56,57^.

## Th9 cells

Th9 cells represent a distinct subset of CD4⁺ T helper cells characterized by their robust production of IL-9, which is considered their signature cytokine^15,22^. Th9 differentiation is primarily driven by the combined signaling of IL-4 and TGFβ, leading to the secretion of cytokines such as IL-9, IL-10, and IL-21^15,22^. This differentiation process is associated with the transcription factors PU.1 and IRF4, which contribute to the establishment of the Th9 cytokine program^22^. However, although these factors are established as master regulators of Th9 cells in general immune contexts, their specific expression and regulatory roles within the periodontal microenvironment remain unvalidated. IL-9 is a pleiotropic cytokine that plays a crucial role in inflammatory responses, immune cell activation, and tissue remodeling through its effects on mast cells, neutrophils, and other immune populations^58^.

Emerging T helper subsets, particularly Th9 cells, have been recognized as potentially important contributors to oral mucosal immune responses. Clinical studies have reported elevated levels of IL-9 in the serum and saliva of patients with gingivitis and periodontitis compared with healthy controls^59,60^, suggesting an association between the IL-9–Th9 axis and inflammatory activity in periodontal tissues. However, currently available evidence remains primarily correlative, and direct causal evidence demonstrating that Th9 cells actively drive periodontal tissue destruction or alveolar bone loss remains lacking. Given its pleiotropic effects on immune regulation and tissue remodeling, IL-9 may exert both pro-inflammatory and immunoregulatory functions depending on the local microenvironment and disease context. Accordingly, elevated IL-9 levels may reflect either disease-promoting inflammatory activity or compensatory immunoregulatory responses to ongoing periodontal inflammation.

## Th17 cells

Th17 cells are a distinct subset of CD4‌^+^ T cells defined by their production of pro-inflammatory cytokines, such as IL-17A, IL-17F, IL-21, and IL-22, with retinoic acid receptor-related orphan receptor gamma t (RORγt) serving as the master transcription factor of Th17 cell lineage^15,61^. Th17 differentiation and expansion are driven by inflammatory cytokines, including IL-6, IL-1β, and IL-23, which are produced by activated antigen-presenting cells in response to dysbiotic microbial stimulation^62^. In particular, IL-23 is considered critical for the stabilization and pathogenic amplification of Th17 responses within chronically inflamed periodontal tissues^32^. Recent evidence has established Th17 cells as central pathogenic drivers of inflammatory bone loss in periodontitis^32,62^. Elevated levels of Th17-associated markers, RORC2 (the human homologue of the master transcription factor RORγt) and IL-17A, have been documented in periodontal lesions compared with healthy gingival tissues, indicating local Th17 accumulation in disease sites^63^. Consistent with these observations, animal models of periodontitis demonstrate a marked expansion of Th17 cells that is closely associated with alveolar bone loss, underscoring their pathogenic contribution^64^. Notably, these Th17 markers are predominantly localized within the lamina propria among infiltrated inflammatory cells, and their expression levels positively correlate with the severity of clinical attachment loss and bone destruction^32^.

In periodontitis, Th17 cells orchestrate periodontal tissue destruction primarily through the secretion of IL-17. This cytokine acts on mesenchymal cells, including osteoblasts and periodontal ligament cells, to upregulate the expression of receptor activator of nuclear factor-κB ligand (RANKL) while concurrently downregulating its decoy receptor osteoprotegerin (OPG), thereby promoting osteoclastogenesis and alveolar bone resorption^32,62,65^. In addition, IL-17 enhances neutrophil recruitment by inducing the production of chemokines such as CXCL1 and CXCL2, as well as granulopoietic factors including granulocyte colony-stimulating factor, leading to amplified inflammatory cell infiltration within periodontal tissues^19^. In this context, excessive neutrophil accumulation and dysregulated activation result in the excessive production of MMPs, reactive oxygen species, and neutrophil extracellular traps, which markedly contribute to periodontal immunopathology and connective tissue breakdown^66–69^.

Collectively, accumulating evidence demonstrates that IL-23 plays a critical role in promoting IL-17 production and sustaining Th17-driven pathology in experimental mouse models and in patients with periodontitis^32,70^. This observation indicates that microbiome-driven IL-23 production contributes to the initiation and amplification of pathogenic Th17 responses, thereby promoting mucosal inflammation and alveolar bone destruction^62,71^. Moreover, it is also pivotal for their expansion, stabilization, and acquisition of a pathogenic phenotype^72^.

Overall, while Th17 responses are primarily pro-inflammatory, IL-17 also protects barrier defenses and host immunity against extracellular microbes, underscoring its context-dependent role in periodontal tissues^27,73^. At controlled or physiological levels, IL-17 contributes to the maintenance of mucosal barrier homeostasis and facilitates efficient microbial clearance without eliciting destructive inflammation^27,73,74^. Thus, a balanced IL-17 response serves to contain infection while preserving tissue integrity, whereas excessive or dysregulated IL-17 production shifts the response toward pathogenic inflammation and tissue destruction.

Beyond the adaptive Th17 cell compartment, innate lymphoid cells (ILCs) are increasingly recognized as vital components of the periodontal immune landscape^75^. In particular, group 3 ILCs (ILC3s) function as the innate counterparts to Th17 cells, sharing the capacity to produce signature pro-inflammatory cytokines such as IL-17A and IL-17F^76^. Similar to Th17 cells, ILC3s are stimulated by cytokines such as IL-1β and IL-23 to initiate pro-inflammatory circuits within the oral mucosa^77,78^. Emerging evidence suggests that IL-23-driven activation of these ILCs contributes to early mucosal immunopathology and neutrophil recruitment^79,80^, potentially exacerbating bone destruction before the full engagement of the adaptive Th17 response. Incorporating the ILC3–Th17 axis thus provides a more comprehensive understanding of the complex lymphoid-driven networks that govern chronic inflammatory destruction in periodontitis.

## Th22 cells

As with Th9 cells, the precise nature of Th22 responses remains unclear. However, the effector cytokine of Th22 cells, IL-22, reportedly mediates diverse functions, including pro-inflammatory, antimicrobial, and wound-healing activities^81,82^. Importantly, although some evidence suggests that IL-22 facilitates mineralized matrix-forming activities in periapical lesions^83,84^, periodontitis remains a distinct pathological entity. In accordance with these findings, IL-22–Th22-related signaling pathways may contribute to tissue stability in both human and experimental periapical lesions^85^. However, these potentially reparative or protective effects observed in periapical tissues should not be directly extrapolated to the immunopathogenesis of periodontitis. The role of IL-22 appears to diverge depending on the inflammatory context and disease stage. Conversely, patients with periodontitis exhibit notably higher levels of IL-22 as well as increased expression of Th22-associated transcription factors, Spi-B and aryl hydrocarbon receptor (AhR), compared with individuals with gingivitis or healthy controls^86^. Although these findings establish a strong clinical association between IL-22-producing Th22 cells and periodontitis, they do not inherently demonstrate that Th22 responses are primary drivers of periodontal pathology.

To distinguish correlation from causality, evidence from functional experimental models is essential. Consistently, experimental mouse models of periodontitis have shown that the increased detection of CD4^+^ AhR^+^ IL-22^+^ T cells is associated with elevated RANKL expression and exacerbated alveolar bone loss^87^. These experimental findings provide mechanistic support for the concept that IL-22-producing Th22 cells may actively participate in RANKL-mediated osteoclastogenic pathways during periodontal inflammation.

Together, these findings suggest that increased levels of IL-22-producing Th22 cells are associated with the pathogenesis of periodontitis, particularly with osteoclast-mediated bone resorption and disease severity. Nevertheless, the independent pathogenic contribution of Th22 cells remains incompletely resolved because IL-22 signaling substantially overlaps with the broader Th17 inflammatory network. Thus, currently available evidence supports a contributory role for IL-22 in periodontal inflammation, but definitive causal evidence establishing Th22 cells as autonomous pathogenic effectors remains limited. Further studies using loss-of-function experimental designs, such as targeted cell depletion or cytokine neutralization, are required to elucidate the precise context-dependent effects by which IL-22 influences periodontal inflammation and tissue remodeling independently of the Th17–IL-17 axis. In addition, the substantial functional overlap between Th17 and Th22 responses further highlights the plastic and interconnected nature of pathogenic T cell programs within periodontal inflammatory lesions.

## Tfh cells

Tfh cells represent a specialized subset of CD4⁺ T cells that are essential for helping B cells and mediating humoral immune responses^24,83,88^. Their differentiation is primarily driven by cytokines, such as IL-6 and IL-21, leading to the expression of the master transcription factor Bcl6^15,89^. Tfh cells are characterized by high expression of the chemokine receptor CXCR5, which facilitates their migration into B cell follicles, as well as surface markers including ICOS and PD-1^83,88^.

In periodontitis, Tfh cells play a critical role through their interaction with B cells and plasma cells, which are prominently enriched in diseased gingival tissues^88,90^. The signature cytokine produced by Tfh cells, IL-21, is vital for germinal center formation, B cell proliferation, and immunoglobulin isotype switching^64,90–92^. Emerging evidence further suggests that Tfh cells contribute to periodontal immune regulation not only through B cell activation but also through lineage plasticity. Recent studies have reported that Th17 cells can acquire Tfh-like features during chronic inflammation, potentially influencing local humoral immune responses^93^. However, the functional significance and pathogenic consequences of Th17-to-Tfh plasticity in periodontitis remain incompletely understood.

Although Tfh cells are relatively rare within periodontitis and related chronic oral inflammatory lesions, emerging evidence suggests that they play a role in regulating local B cell responses and antibody production^83,93^. Importantly, currently available evidence remains largely based on marker expression profiling and observational association studies rather than functional intervention models. Therefore, direct mechanistic evidence defining how Tfh cells regulate periodontal tissue destruction, osteoclastogenesis, or alveolar bone resorption remains limited.

Specifically, clinical studies have identified the expression of Tfh cell markers in periapical lesions, where they likely participate in the inflammatory cytokine networks that determine lesion activity^83,85,92^. However, because periodontitis is a distinct pathological entity, these findings should not be directly extrapolated to periodontal immunopathogenesis without caution. Understanding the Tfh–B cell axis is therefore increasingly essential for a comprehensive view of the adaptive immune landscape in periodontal disease.

Collectively, these observations support the emerging concept that chronic periodontal inflammation promotes dynamic interconversion among T helper subsets, particularly along the Th17–Tfh axis, thereby reshaping local adaptive immune responses during disease progression.

## Treg cells

Treg cells represent a pivotal immunoregulatory subset of CD4⁺ T lymphocytes that play a central role in maintaining immune homeostasis and preventing excessive inflammatory responses^15,94,95^. Treg cells are characterized by expression of the transcription factor forkhead box P3 (Foxp3), high levels of CD25 (IL-2 receptor α chain), and low expression of CD127. Treg cells suppress immune activation through both cell contact-dependent mechanisms and the secretion of anti-inflammatory cytokines, including TGFβ, IL-10, and IL-35^15,96,97^. In periodontal disease, Treg cells are increasingly recognized as critical modulators of host–microbe interactions and inflammation-driven tissue destruction^96,98^. Notably, in the inflammatory milieu of periodontitis, Treg cells act as key suppressors that restrain Th1-, Th17-, and B cell-mediated inflammation^88,96,98^.

Foxp3⁺ Treg cells have been detected in human gingival tissues affected by periodontitis, and experimental models of periodontitis^99,100^. Their presence is generally interpreted as a protective host response aimed at restraining excessive inflammation^100,101^. Mechanistically, Treg cells suppress effector T cell proliferation and cytokine production, thereby limiting osteoclastogenesis through indirect pathways. Specifically, IL-10 and TGFβ produced by Treg cells can attenuate the expression of RANKL in effector T cells and mesenchymal cells, which in turn reduces the pro-resorptive signaling necessary for osteoclast differentiation^89,101,102^. This regulatory influence on the local cytokine milieu serves to shift the RANKL–OPG balance toward a more bone-protective state, thereby reducing alveolar bone resorption. Furthermore, Treg-mediated suppression of Th17 responses is particularly relevant, as IL-17 has been strongly implicated in periodontal bone destruction^103^. Recently, experimental evidence from animal models support a protective role for Treg cells in periodontal disease^104,105^. Expansion or adoptive transfer of Treg cells reportedly reduces inflammatory cytokine production and alveolar bone loss, whereas depletion of Treg cells exacerbates periodontal inflammation and osteoclast activity^89,104,105^. These findings highlight Treg cells as potential therapeutic targets for host modulation strategies aimed at controlling destructive inflammation without compromising antimicrobial defense.

In summary, Treg cells serve as pivotal regulators of immune responses in periodontal disease. By constraining excessive inflammation and modulating osteoclastogenesis, they contribute to the maintenance of periodontal tissue homeostasis. However, accumulating evidence suggests that Treg cells in periodontitis undergo functional instability and acquire pro-inflammatory characteristics under chronic inflammatory conditions. This plasticity within the Treg compartment may contribute to the breakdown of immune homeostasis and facilitate sustained periodontal tissue destruction.

## Plasticity and imbalance of T cells in periodontitis

Periodontal disease has been widely interpreted through a Th1–Th2 paradigm, in which Th1 responses were associated with pro-inflammatory tissue pathology, whereas Th2 responses were considered protective or regulatory^106^. However, accumulating evidence since the early 2010s has challenged this simplified dichotomy, revealing a far more complex immune landscape within periodontal lesions^4,34,56^. In the periodontal lesion milieu, Th1 and Th2 cytokines are simultaneously detected, reflecting a complex and interconnected immune network rather than a simple dichotomous polarization^56,107^. Both Th1 and Th2 responses coexist in periodontal disease, suggesting a multifaceted immune milieu rather than a simple Th1–Th2 dichotomy^56^. Importantly, this concurrent expression indicates that periodontal disease is not governed by a unidirectional T helper cell response^44,56^. Instead, the relative dominance of Th1 or Th2 activity appears to fluctuate depending on the disease stage, microbial burden, and local cytokine milieu^44,57^.

Beyond the balance between subsets, T helper cell plasticity and functional adaptation have emerged as important concepts in understanding periodontal immunopathogenesis^56,108^. CD4⁺ T cells exhibit substantial functional flexibility, enabling Th1 and Th2 cells to modulate their cytokine expression profiles in response to environmental cues such as antigen load, cytokine gradients, and cellular interactions within the periodontal microenvironment^107,108^. For example, persistent IL‑10-mediated regulatory signaling can induce a shift in Th1 cells^109^ that downregulates their IFNγ production via IL‑10-dependent feedback mechanisms during periodontal disease^43^. Conversely, Th2 cells within periodontal lesions may upregulate not only classical cytokines, such as IL‑4 and IL‑13, but also additional inflammatory mediators in response to pro‑inflammatory cues, reflecting broader functional flexibility within disease-associated microenvironments^43,44^. These observations suggest context-dependent functional modulation of Th2 responses rather than fixed effector programs, highlighting the dynamic nature of T helper cell regulation within periodontal lesions.

Therefore, periodontal disease should not be understood as a condition driven by a simple reciprocal imbalance between Th1 and Th2 responses, but rather as a dynamic process shaped by continuous functional recalibration among CD4⁺ T cell subsets. Periodontal inflammation represents an evolving immune response that adapts to fluctuating microbial challenges and host-derived regulatory mechanisms. Recognizing this complexity is essential for developing immunomodulatory strategies that restore immune homeostasis rather than selectively suppress a single T helper cell lineage.

**Fig. 1:**
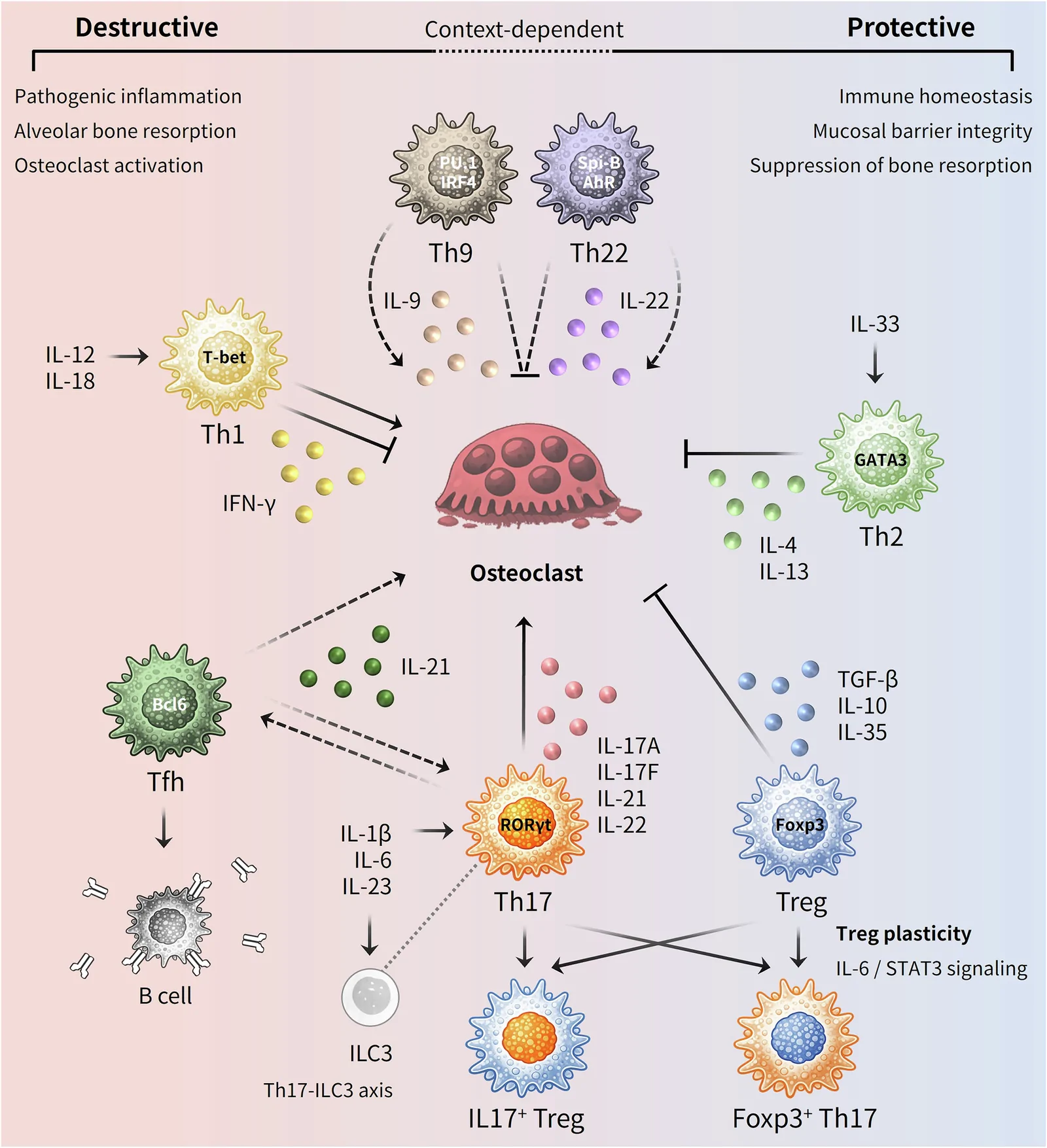
Mechanistic model of T cell plasticity and Th17–Treg imbalance in periodontal tissue destruction. Illustration of the mechanisms linking dysbiotic microbiota to Th17–Treg imbalance, T cell plasticity, chronic inflammation, and alveolar bone loss in periodontitis. Pro-inflammatory cytokines promote Th17 responses and destabilize Treg cells, resulting in enhanced IL-17 production, inflammatory mediator release, osteoclastogenesis, and periodontal tissue destruction. *CXCL1–CXCL2* C-X-C motif chemokine ligand 1 and 2, *DCs* dendritic cells, *G-CSF* granulocyte colony-stimulating factor, *ILC3* group 3 innate lymphoid cells, *MMPs* matrix metalloproteinases, *NETs* neutrophil extracellular traps, *OPG* osteoprotegerin, *PDL* periodontal ligament, *RANKL* receptor activator of nuclear factor-κB ligand, *ROS* reactive oxygen species, *STAT3* signal transducer and activator of transcription 3.

Among these dynamic T cell networks, the balance and plasticity between Th17 and Treg cells are particularly important determinants of periodontal inflammatory bone destruction. The proposed mechanisms linking dysbiotic microbial stimulation, Th17 expansion, Treg instability, and inflammatory bone loss are summarized in Fig. 1.

As described above, Th17 cells represent a subset of CD4⁺ T cells that promote osteoclast differentiation, whereas Treg cells suppress inflammatory responses and inhibit osteoclastogenesis. However, this balance becomes disrupted in periodontitis, resulting in a relative dominance of Th17 responses over Treg-mediated immunoregulation. Among them, some studies emphasized that the balance between Th17 and Treg cells plays a pivotal role in the regulation of inflammatory bone metabolism^103,110,111^. Consistently, an increased Th17/Treg ratio has been observed in periodontal lesions and peripheral blood of patients with periodontitis, correlating with disease severity and progressive alveolar bone loss^112^.

**Fig. 2:**
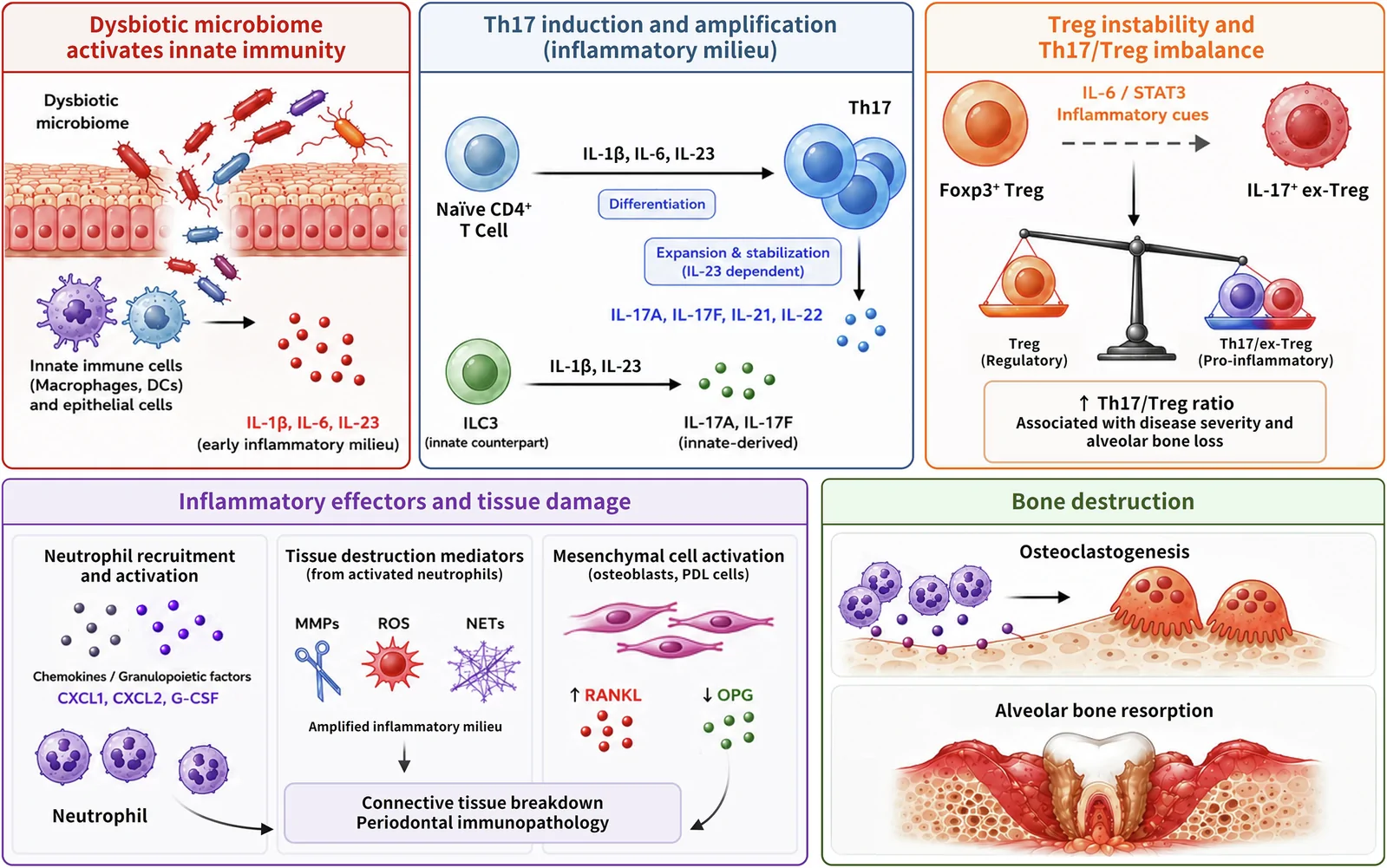
Plasticity and context-dependent functions of CD4^+^ T helper cell subsets in periodontitis. Schematic overview of major CD4^+^ T helper cell subsets involved in periodontitis and their context-dependent roles in periodontal inflammation and tissue homeostasis. The figure illustrates the balance between destructive and protective immune responses, including Th17–Treg imbalance, T cell plasticity, and interconversion among T helper subsets within the periodontal microenvironment. *AhR* aryl hydrocarbon receptor, *Bcl6* B cell lymphoma 6, *Foxp3* forkhead box P3, *GATA3* GATA-binding protein 3, *IFNγ* interferon-γ, *IL* interleukin, *ILC3* group 3 innate lymphoid cell, *IRF4* interferon regulatory factor 4, *PU.1* Spi-1 proto-oncogene, *RORγt* retinoic acid receptor-related orphan receptor gamma t, *STAT3* signal transducer and activator of transcription 3, *TGFβ* transforming growth factor-β.

Although increased Treg infiltration may limit tissue damage, excessive immunosuppression could theoretically impair effective antimicrobial immunity, allowing persistence of periodontal pathogens^96,98^. However, Treg cells play a complex and context-dependent role in periodontitis^104^. The plasticity of CD4⁺ T cells further complicates this landscape, as inflammatory cytokines such as IL-6 can destabilize Foxp3 expression and shift the balance toward pathogenic Th17-like phenotypes^104^. Chronic IL‑6–STAT3 signaling destabilizes Foxp3 expression by altering TSDR methylation, predisposing Treg cells toward conversion into IL‑17-producing ex‑Treg cells^113^. Notably, recent studies demonstrate that a subset of Foxp3⁺ Treg cells can convert into IL‑17-producing ex‑Treg cells under inflammatory conditions, contributing to pathogenic IL‑17 pools in periodontal lesions. Emerging evidence has identified the presence of CD4⁺ T cells exhibiting mixed phenotypes, such as IL-17-producing Treg cells (IL-17⁺ Treg) or Foxp3-expressing Th17 cells (Foxp3⁺ Th17)^66,103,104,114^. These hybrid populations display a markedly enhanced osteoclastogenic potential compared with conventional Th17 cells and have been implicated as key contributors to pathological bone resorption under chronic inflammatory conditions, including periodontitis (Fig. 2).

Beyond cellular phenotypes and ratios, recent advances in spatial transcriptomics and tissue mapping have revealed that the periodontal immune landscape is highly organized. Specifically, recent spatial transcriptomic and single-cell studies suggest the presence of highly organized immune niches, including immune cell aggregates with features resembling tertiary lymphoid architectures, in periodontitis lesions^115,116^. These structures function as localized immunological hubs that sustain antigen presentation, T cell activation, and B cell maturation directly within chronically inflamed gingival tissues.

Importantly, the spatial proximity between T helper cells and B cell-rich compartments may facilitate persistent cytokine signaling, Tfh-associated humoral responses, and the stabilization of chronic inflammatory circuits that become resistant to systemic immune regulation. In this context, the periodontal microenvironment is increasingly recognized not merely as a passive site of inflammation but as an active regulator of T cell functional plasticity, lineage stability, and immune persistence.

These findings provide important mechanistic insight into how cellular localization and microenvironmental context shape T cell behavior during periodontitis progression. Incorporating the concept of tertiary lymphoid architectures into the periodontal T cell framework therefore emphasizes the critical importance of spatial immune organization as a driver of sustained inflammation, pathogenic immune remodeling, and immune-mediated alveolar bone loss.

The close spatial organization of antigen-presenting cells, T helper cells, and B cell-rich compartments may facilitate sustained exposure to inflammatory cytokines such as IL-6 and IL-23, which influence Th17 expansion and Treg instability. Although direct evidence remains limited, these observations suggest that spatial immune organization contribute to the maintenance of pathogenic immune states within periodontal lesions.

Collectively, these findings suggest that dynamic Th17–Treg conversion occurs within the highly organized periodontal inflammatory microenvironment, underscoring the Th17–Treg axis as a key determinant of periodontal disease progression. Emerging evidence further indicates that spatially organized inflammatory niches within periodontal lesions may contribute to the regulation of T cell phenotypes and immune responses. Therefore, a deeper understanding of Th17 and Treg stability, plasticity, and interactions within the periodontal microenvironment may inform the development of next-generation immunomodulatory strategies aimed at restoring immune homeostasis and limiting inflammatory bone loss in periodontitis.

## Conclusion

Periodontitis is a chronic inflammatory disease initiated by a dysbiotic microbiome; however, the majority of tissue damage results from an exaggerated host immune response rather than direct bacterial cytotoxicity. Within periodontal lesions, a complex and dynamically regulated network of innate and adaptive immune cells, including Th1, Th17, and B cells, contribute to the production of pro-inflammatory mediators, such as IL-1β, TNFα, IL-6, and IL-17, which collectively promote osteoclastogenesis and connective tissue breakdown.

Counterbalancing these pathogenic responses, Treg cells function as a critical immunosuppressive population that restrains excessive inflammation and limits immune-mediated tissue injury. Nevertheless, accumulating evidence indicates that periodontal inflammation is not governed by a simple dichotomy between destructive and protective T cell subsets. Rather, disease progression reflects a dynamic and context-dependent recalibration of CD4⁺ T cell responses, shaped by microbial burden, local cytokine gradients, and cellular interactions within the periodontal microenvironment (Fig. 1).

The plasticity of T helper cells, including the emergence of hybrid phenotypes such as IL-17⁺ Treg cells or Foxp3⁺ Th17 cells, underscores the dynamic yet unstable nature of immune regulation in periodontitis and illustrates how maladaptive reprogramming can enhance osteoclastogenic potential and pathological bone resorption. Accordingly, periodontitis should not be viewed as the outcome of a single dominant T cell lineage. Instead, it represents the manifestation of an evolving immune network in which imbalance and phenotypic instability of CD4⁺ T cells determine tissue fate. In this context, inflammatory bone loss reflects not only the expansion of pathogenic subsets, but also the erosion of coordinated regulatory mechanisms that normally sustain mucosal homeostasis.

Recognition of T helper cell plasticity challenges the traditional strategy of selectively targeting individual cytokines or discrete T cell lineages. Therapeutic approaches focused solely on suppressing specific pro-inflammatory mediators may be insufficient if the underlying immune dysregulation persists. Future interventions should instead aim to reinforce regulatory stability, preserve protective T cell phenotypes, and restore a balanced cytokine environment that supports controlled host–microbe equilibrium.

Ultimately, advancing our understanding of T cell plasticity, phenotypic instability, and immune adaptation within the periodontal microenvironment will be essential for designing next-generation precision host modulatory therapies capable of restoring durable immune homeostasis, preventing pathological osteoclastogenesis, and maintaining periodontal tissue integrity without impairing antimicrobial defense.

## Data Availability

Data sharing is not applicable to this article as no new data were created or analyzed in this study.
